# Neoplastic Pathogenesis Associated with Cigarette Carcinogens

**DOI:** 10.7759/cureus.3955

**Published:** 2019-01-25

**Authors:** Azhar Hussain, Parmvir Dulay, Megan N Rivera, Carla Aramouni, Vishal Saxena

**Affiliations:** 1 Epidemiology and Public Health, Xavier University School of Medicine, Oranjestad, ABW; 2 Internal Medicine, Xavier University School of Medicine, Oranjestad, ABW; 3 Pathology, Xavier University School of Medicine, Oranjestad, ABW

**Keywords:** cigarette, carcinogenic agent, neoplasms, tobacco

## Abstract

Cigarette smoke is widely regarded as a carcinogenic agent; thus, the incidence of relative neoplasms correlates to cigarette smoking (CS) on a global level. While CS is most commonly associated with carcinomas of the upper and lower respiratory tracts, studies have also associated CS with the pathogenesis of a variety of non-respiratory related neoplasms. The tobacco smoke emitted from cigarettes contains carcinogenic substances that can be harmful to the normal physiology of the human body. This study will elaborate on the incidence and etiology of carcinomas, as well as discuss, in detail, the role of tobacco in the pathogenesis of oral, esophageal, lung, gastric, pancreatic, renal, and bladder carcinomas.

## Introduction and background

The epidemic of cigarette smoking (CS) is one of the largest public health threats and one of the leading causes of preventable deaths, globally. Tobacco kills more than seven million people every year [1], out of which 480,000 deaths per year are in the United States (U.S.) alone [2]. There are over 7,000 chemicals in tobacco smoke, and more than 250 of those toxins are known to be carcinogenic. These include polycyclic aromatic hydrocarbons (PAHs), cadmium and beryllium (toxic metals), hydrogen cyanide, carbon monoxide, and ammonia [3]. There are many forms of tobacco in the market, including cigars, electronic cigarettes, hookah (water pipe), as well as bidis, which are flavored cigarettes. However, there is no safe form of tobacco, and all of them can cause severe health problems, which include neoplasms, cardiovascular issues, and respiratory problems, such as obstructive and restrictive lung disease [2-3]. Approximately 40% of all cancers diagnosed in the U.S. are due to the use of tobacco [4]. The dose, type of tobacco, route of nicotine delivery, and chronicity of irritation exposure to the cancer-prone area are all determinants of cigarette smoke on the health outcome of an individual [5].

Studies have shown that the risk of cancer is directly proportional to the number of cigarettes smoked per day and the duration of chronic smoking [6]. However, each cigarette contains approximately 10 milligrams of nicotine, which is a highly addictive substance [3,6]. Currently, about 37.8 million adults smoke in the U.S. [2]. Out of those, 70% would like to quit but are not able to do so due to withdrawal symptoms, including irritability, depressed mood, restlessness, and anxiety [3]. 

Recent studies have shown that CS may lead to cancers in the oral cavity [7], esophagus [8], lung, stomach [9], pancreas [10], kidneys [11], and the bladder (bladder cancer; BC) [12]. The carcinogens found in the cigarettes may lead to DNA damage [13], impaired DNA repair mechanism [14], and a weakened immune system, which further leads to inflammation and infections [11]. CS causing impaired immunity in the oral cavity ultimately leads to oral cancer (OC) [7]. Over time, the superficial ulceration of a mucosal surface in the oral cavity develops, and it may become an exophytic mass (EM), or tumor, which tends to grow outward beyond the surface epithelium from which it originates [15]. If the initial ulceration, or lesion, is left untreated or the irritant is not removed, it can progress to carcinoma of the surrounding tissues, as in oral squamous cell carcinoma (OSCC) [16]. By contrast, in the development of esophageal squamous cell carcinoma (ESCC), through a multi-step process of epigenetic and genetic changes, a sequence of histological changes occurs in the epithelium of the esophagus, over years of chronic irritation due to tobacco smoking [17]. Studies have shown that the nicotine from smoking may increase the gastric aggressive factors and attenuate the gastric defense mechanism, which results in gastric ulcers (GUs) and leads to gastric adenocarcinoma (GAC) [18]. The exact etiology of renal cell carcinoma (RCC) is unknown; however, there are many known risk factors, which include obesity, hypertension (HTN), family history, advanced kidney disease, and CS [19]. CS is the major cause of urothelial cell carcinoma (UCC) as well [12]. This article focuses on CS-associated cancers and their pathogeneses.

## Review

Oral cancer associated with CS

Squamous Cell Carcinoma of the Oral Cavity

The Centers for Disease Control and Prevention (CDC) reported that in 2012, there were nearly 40,000 new cases of cancer involving the oral cavity and pharynx, which resulted in nearly 9,000 deaths in the U.S. and was deemed the sixth most common cancer worldwide [20-21]. Tobacco use in the form of smoking cigarettes, smokeless tobacco, cigars, and chewing tobacco directly affect the oral cavity, which may lead to OSCC [7]. The oral cavity is the first site exposed to the carcinogens found in tobacco products, inevitably resulting in an increase in incidences of OC [5].

The effect of CS on human immunity depends on multiple variables, including the dose and type of tobacco, the route, and chronicity of the exposure [5,7]. Cigarettes deregulate the immune response against pathogens and antigen presentation, therefore promoting autoimmunity throughout the body [7]. The deregulation of the immune response by a heightened or suppressed inflammation (due to CS) in the mucosal surface and the modulated microvasculature affects healing and resolution of inflammation [5,7]. Consequently, impairment of the oral cavity immune response serves to promote OC [7].

The OSCC may arise anywhere within the oral cavity. The most common location is the ventral surface of the tongue, the floor of the mouth, lower lip, soft palate, and the gingiva [16]. Early OSCC presents as leukoplakia, which may lead to erythroplakia, or even erythroleukoplakia, which is a mixture of red and white lesions on the tongue or mucosal surface [15]. Leukoplakia has no histopathological connotation and is not used as a microscopic diagnosis; however, it is instead used as a marker to take a biopsy for further investigations [7,15]. Over time, superficial ulceration of the mucosal surface develops, and it may become an EM [15], and if left untreated, or the irritant is not removed, it can progress to OSCC [16]. The most common site for the cancer is the tongue, on the posterior-lateral border, or on its ventral surface [15]. The lateral tongue and floor of the mouth combine to form a region of the oral mucosa that includes the tonsillar area and the lateral soft palate [7,15]. This region has a high-risk factor because many of the tobacco carcinogens mix with saliva and then pool at the bottom of the mouth; this leads to chronic irritation of this site [7,15,22]. Furthermore, this region of the mouth is covered by a thin, nonkeratinized mucosa that provides less protection against carcinogens [15,22]. OSCC has a risk of metastasis; when the cancer cells enter the vascular or lymphatic channels and into the cervical nodes, metastasis can occur [21]. The most common areas of metastasis are the lungs, lymph nodes, liver, and bones [15,21].

Treatment options are similar to the treatment of other cancers, which are variable, and depend on the following: the size and location of the primary tumor, lymph node status, and the presence or absence of metastases. Surgery and/or radiation therapy is the gold standard for cancers of the lip and oral cavity [23]. For advanced stages, surgery with radiation therapy may be warranted [21,23].

Esophageal cancer associated with CS

Squamous Cell Carcinoma of the Esophagus

EC has become an increasingly common and lethal malignancy. According to the CDC, “a total of 81,608 new cases (4.6 per 100,000 people) of EC were reported in the U.S. during 2010-2014” [24]. ESCC is the most common type of EC globally [8,24]. The incidence of ESCC increases with age, occurring mostly in the seventh decade of life, and most often in the middle and lower esophagus [17]. The high-incidence area for ESCC is associated with the “Asian EC belt,” which is from Central China, East Asia, into Iran [8,17,24]. In the high-incidence area, about 90% of the ECs are ESCC, versus in North America, in which less than 30% are [8,17]. The leading risk factors for ESCC in Western countries is tobacco smoking [8]. Studies have shown that chronic tobacco smokers have a relative risk of 2.5-4 folds of developing ESCC than those who have never smoked tobacco products [25].

Recent studies have shown that having tobacco exposure at a young age, or with high intensity and accumulative consumption, further increases the risk of developing ESCC [17,25]. The long-term irritation of the esophagus by the carcinogens in tobacco smoke has a damaging effect on the DNA in the squamous cells that line the inside the esophagus. Cigarette smoke contains free radicals and induces oxidative damage [14]. The damaging effect on DNA from the constant irritation by the carcinogens affects the cytochrome 450 (CYPs) enzymes. The CYPs catalyzes an oxygen atom onto a carcinogen, to increase its water solubility and convert into a form that is readily excretable. However, with persistent cigarette smoking, some of the intermediates (the carcinogens) that are formed by the interaction with CYPs are reactive, and when react with DNA, result in DNA adducts [25]. DNA has an elaborate repair system to eliminate DNA adducts from the genome; however, if the repair mechanism fails, due to irritant overload such as tobacco use, it can lead to mutations, which increases the risk of cancers [14].

The ESCC develops through a multi-step process of epigenetic and genetic changes, leading to a sequence of histological changes in the epithelium of the esophagus over years of chronic irritation, in this case by tobacco smoking [17]. The histological changes of the esophagus begin with esophagitis, then basal cell hypertropia, dysplasia, and carcinoma in situ, ultimately leading to ESCC [8,25]. The esophagus lacks serosa and without an anatomical barrier, the primary tumor can spread rapidly into adjacent structures of the neck and thorax [17]. The prognosis of ESCC depends on the local invasion, as well the spread to regional and distant structures within the body. ESCC has a high risk of invading the thyroid gland, trachea, larynx, lung, pericardium, aorta and diaphragm [8]. Once ESCC has invaded the surrounding structures, a patient presents with dysphagia for liquids at first, and consequently experiences progressive dysphagia including solid food, due to the growth of cancer [17]. As a result, odynophagia occurs due to the obstruction by cancer, resulting in weight loss, due to lack of nutrition [8,17]. Other complications that may occur, depending on the location of cancer, may include the following: bleeding, epigastric pain, hoarseness, persistent cough as well as respiratory symptoms, such as difficulty breathing. However, patients may remain asymptomatic until malignancies have already occurred [17]. 

ESCC is known to be aggressive by nature through its spread by a variety of pathways including the lymphatic system and hematogenous metastasis [8,17]. The lymphatic drainage of the esophagus is by two separate lymphatic plexuses. One arises within the mucosal layer and the other arises from the muscular layer. The lymphatic fluid from the upper two-thirds of the esophagus drains up, while the lymph from the lower one-third of the esophagus drains down; however, all the lymphatic channels of the esophagus still communicate [14,17]. The lymphatic fluid from any portion of the esophagus may spread into the intra-thorax or intra-abdominal lymph nodes, resulting in metastases [8,17].

The prognosis for patients diagnosed with ESCC is poor. Approximately 10% of patients survive five years after the diagnosis; thus, the approach of patient care is primarily focused on the management of controlling symptoms. Current treatment options include surgery, radiation, and chemotherapy [17].

Lung cancers associated with CS

Small Cell Lung Carcinoma

SCLC is a carcinoma of the lungs, which is very aggressive and has rapid growth and an early metastasizing pattern [26]. About every two-thirds of the people, when already diagnosed with SCLC, have this carcinoma in its extensive stages [27]. This carcinoma arises from the neuroendocrine cells and is known as “Oat Cell Carcinoma” due to its appearance. On the microscope, this carcinoma appears pale and grayish, while the cells are oval-shaped, therefore resembling an oat [26-27]. These cells are highly malignant and have a very thin cytoplasm and fine granular chromatin. SCLC is a centrally located mass of the lungs. It has an early metastasizing effect into the lung parenchyma and most commonly involves the hilar and mediastinal lymph nodes. SCLC accounts for about 10% to 15% of all LCs [28]. There have been a total of 234,030 new cases and 154,050 deaths due to LC in the U.S. alone. The good news is that since the smoking rates have been declining over the past few years, the incidence of SCLC is decreasing. The manifestation usually occurs around the age of 60-80 years and most commonly affects males. This is mainly due to the fact that smoking is the leading cause of SCLC and makes up about 98% of cases [9]. Epidemiological studies have shown that males generally smoke more than females; hence, the risk of developing SCLC is much greater in males than females. Another reason is that males tend to more often work in factories and are therefore exposed to chemicals like radon, asbestos, and uranium, which puts them at a higher risk [9,27-28]. SCLC more commonly arises in the later decades of life which is due to the fact that smokers more often have had exposure to smoking their entire lives. This demographic causes gradual damage to their lungs over time while allowing for an adequate recovery period for their lungs to regenerate from the lifelong effects of cigarette smoke [9,27].

Since the most common risk factor for SCLC is smoking, it is rare to see a patient present with a different cause. Some other risk factors include exposure to other harmful chemicals, second-hand smoke, and a family history of SCLC [27]. Any type, whether it may be cigarette smoke, pipe smoking, cigar, electronic CS, and even low-tar cigarettes, are all just as likely to cause SCLC. The longer one smokes and the greater the amount that is smoked will each be a factor in how much higher the chance of developing SCLC is. If smoking is stopped, the probability of survival will likely rise, thus the risk of getting SCLC will improve, or decrease [9].

The development of SCLC depends on the extent of the lung damage due to smoking. Smoking damages the lining of cells in the lungs, which makes the lungs weaker and affects tissue. Although the body can repair damage for the carcinogens that enter it, the healing capacity of the body decreases, which leads to the further damage of tissue. So, over time, the accumulation of tobacco smoke that has harmed the lungs, and irreparable damage leads to diseases like SCLC [9,27]. The development of SCLC also leads to some paraneoplastic syndromes that are associated with it, due to inappropriate secretion of some hormones which can also manifest as other diseases. With cancer, organ and tissue function are affected, and that affects the normal processes of the body. The body, for example, produces excessive amounts of antidiuretic hormone (ADH), leading to SIADH, a condition that causes the body to retain water [9,28]. Another manifestation associated with SCLC is Cushing’s syndrome which is due to excessive secretion of adrenocorticotropic hormone (ACTH) [9]. Most patients are asymptomatic at early stages, but in the advanced stages of SCLC, will present most commonly with shortness of breath, hemoptysis, fatigue and weight loss [9,28].

The most common treatment of SCLC includes a combination of chemotherapy and radiation therapy. Combining of the two will increase the survival rate and prolong the life of the patient because they both work to stop the spread of, and kill, cancer. Chemotherapy works by preventing the disease from spreading, while radiation uses high-energy particles to destroy the cancer cells. Surgery is not recommended due to the fact that this cancer can spread very fast; for even just by the time of diagnosis, resection from only one place will not be sufficient to get rid of cancer [28].

Non-Small Cell Lung Carcinoma

Adenocarcinoma: Adenocarcinomas are non-small cell lung carcinomas (NSCLC) and are the most common cause of primary LC. NSCLCs make up 80% of all LCs and 40% of those are due to adenocarcinomas [26,28], which arise from the glandular cells of the outer portion of the lung, leading to a peripherally located mass [28]. The glandular cells help secrete mucus, which is needed as a protective covering to help fight against foreign particles from entering the lung. It also shows columnar cells that grow along the lines of bronchioles and alveoli. An adenocarcinoma is a slow-growing cancer and is usually diagnosed before it spreads, which makes its prognosis better than most LCs [4].

There have been 218,527 new cases of lung and bronchus cancer in the U.S. reported as of 2015, and 153,718 resulted in death [20]. Although rates of LC are slowly dropping, adenocarcinoma is found to be the most common type of LC found in females. Female prevalence may be due to the fact that women began smoking later in life compared to men and then continued to smoke when men began to stop [28].

The adenocarcinoma is the most common LC in non-smokers, even though smoking is one of its risk factors. Smoking damages the cell lining of the lungs. This harm over time causes the lungs to lose their ability to fix the scarring having occurred on the lung, which is when adenocarcinoma can arise. Adenocarcinoma that has spread can occur due to a mutation in the genes of Kirsten rat sarcoma viral oncogene homolog (K-RAS), epidermal growth factor receptor (EGFR), and anaplastic lymphoma kinase (ALK). A mutation in the gene of K-RAS is most associated with smoking and accounts for about 25% of cases. Meanwhile, EGFR and ALK are not related to smoking [29]. Testing for these gene mutations will ultimately determine the appropriate treatment to go forward with when treating adenocarcinoma; hence, if the K-RAS mutation is present, that would indicate that the anti-EGFR treatment will not work. The anti-EGFR treatment is commonly used in adenocarcinoma of the lung that is spread widely in the body [28-29]. Adenocarcinoma patients present most commonly with hemoptysis, cough, chest pain, weight loss, and fatigue [28].

The treatment options for adenocarcinoma widely depend on the stage of the cancer. If it is still localized and has not metastasized, then surgical resection is the best option. Sometimes chemotherapy is used first to stop the growth and then surgery is used to resect the tumor. Finally, if cancer has spread past the lungs, then chemoradiation would be used to stop the spread and kill cancer cells, followed by surgery if it is needed. Also, if cancer has metastasized, testing for gene mutations will help treat those that require the anti-EGFR treatment followed by surgery [28-29].

Squamous cell carcinoma: The consumption of tobacco products by smoking cigarettes, pipes, and cigars is the leading cause of LC [21]. The American Cancer Society reports that NSCLC, including squamous cell carcinoma of the lung (SCCL), account for about 80% to 85% of LCs [28]. It was previously the most common type of LC, but in many countries, it has declined over the past four decades, with a rise of adenocarcinoma of the lung instead [20], now being the most common. SCCL is seen more frequently in male smokers [30] and arises after decades of exposure to smoking. 

SCCL is preceded by the development of squamous dysplasia or metaplasia in the bronchial epithelium over years [31]. If the irritant is not removed, the squamous metaplasia can transform to carcinoma in situ, and eventually SCCL [28,31]. SCCL arises centrally in the major bronchi as a small neoplasm and eventually spreads to the hilar group of lymph nodes [31]. The small neoplasm becomes symptomatic, and a well-defined tumor mass continues on to obstruct the lumen of a major bronchus, often producing atelectasis and infection [30-31]. Large lesions may undergo central necrosis and give rise to cavitation [31]. SCCL shows keratinization and/or intercellular bridges that arise from the bronchial epithelium, due to the squamous metaplasia [30-31].

SCCL has a high probability of metastases to other parts of the body, due to the constant flow of fluids, such as blood and lymph, through the lungs, which carry cancer cells to nearby areas [32]. A complication of SCCL is the development of Pancoast tumors in the upper lobe, where it can compress blood vessels and nerves [31-32]. This can ultimately damage the thoracic inlet, brachial plexus, and the cervical sympathetic nerve, resulting in Horner syndrome, manifesting as miosis, ptosis, anhidrosis, as well as enophthalmos [30-32]. Treatment of SCCL heavily depends on the stage the cancer is in and the patient’s overall health. Treatment includes surgery, radiation therapy, chemotherapy, and immunotherapy [30].

Gastric cancers associated with CS

Gastric Adenocarcinoma

Gastric adenocarcinoma (GAC) is the fourth most common cancer of the gastrointestinal system and the third leading cause of global cancer-related death. Approximately 1,000,000 individuals are diagnosed with GC annually, resulting in 800,000 deaths a year [33]. The pathogenesis of GC is multifactorial; however, studies have shown that smoking plays a critical role in the manifestation of this cancer. The most common type of GC associated with smoking is GAC. They are classified by Lauren’s criteria into two major histological subtypes, namely the intestinal and diffuse types. In intestinal type adenocarcinoma, tumor cells exhibit adhesion and are arranged in tubular or glandular formation. This type is often associated with intestinal metaplasia and commonly affects the gastric antrum [33-34]. In contrast with intestinal adenocarcinoma (IAC), the diffuse type adenocarcinoma cells lack adhesion infiltrate in the stroma as a single cell or a small subgroup, resulting in a population of non-cohesive, scattered tumor cells. The diffuse type exhibits more aggressive features as compared to the IAC [33].

Although *Helicobacter pylori (H.pylori)* infection is the most common cause, smoking has been found to have increased the risk of GAC. Studies have shown that the nicotine from smoking may increase the gastric aggressive factors and attenuate the gastric defense mechanism, resulting in GUs, which then leads to the GAC. Smoking increases the gastric acid secretion by histamine or pentagastrin, rather than basal acid secretion [18]. The increase in gastric acid decreases the pH of the gastric mucosa, which leads to the activation of pepsin since the activity of pepsin is highly dependent on the gastric pH [33]. The long-term effects of smoking cause the inhibition of the mucus secretion from the gastric epithelium, which is a protective barrier against gastric acid. The combined effects of histamine and pepsin in increasing the gastric acid production, and decreasing the pH, acutely cause erosion of the mucosal layer. This may result in chronic GUs and hemorrhages, which may lead to GAC [18,33].

The reactive oxygen species (ROS) are well documented to be involved in the pathogenesis of gastric inflammation, ulcerogenesis, as well as carcinogenesis [35]. Kalra et al. found that there is a significant increase in ROS activation in smokers compared to non-smokers, suggesting that ROS may promote mucosal injury in smokers [36]. Chronic smoking also stimulates the secretions of vasopressin, which is a potent vasoconstrictor, by activating central nicotinic cholinergic projections to the hypothalamus [37]. The vasopressin causes generalized vasoconstriction, which decreases the blood flow to the gastric mucosa. This results in tissue hypoxia, which leads to gastric injury and a slow healing process [18].

The tumor protein p53 (TP53) gene provides instructions to make tumor protein p53 (or p53), which acts as a tumor suppressor. The p53 protein binds directly to the DNA, and when there is DNA damage, the p53 activates other genes to either repair the DNA damage, or activate the apoptosis pathway, if DNA is not repairable [13]. The TP53 is the most frequently mutated gene in GCs, exhibiting aberrations in approximately 50% of cases [33-34]. The long-term effects of tobacco smoking have been associated with an increased TP53 gene mutation, which impairs the DNA repair mechanism, resulting in abnormal proliferation of the cell, which leads to tumor growth [13].

The treatment includes surgical approach based on the location of cancer, size, and tumor extent. The chemotherapy and radiotherapy can be used, and often time recommended, before surgery to improve the outcome of intended surgery. In advanced cases, palliative care is offered [18,21].

Pancreatic cancers associated with CS

Pancreatic Adenocarcinoma

The pancreas is an organ in the abdomen that is located behind the stomach and is pear-shaped [10-11]. The cancer of the pancreas is known to be highly invasive and has a very strong association to tobacco smoking. PAC starts when cells from the tissue of the pancreas start to grow uncontrollably and become cancerous [10]. The pancreas contributes to both endocrine and exocrine functions, playing a vital role in the body. It secretes the hormones insulin as well as glucagon, which are important in maintaining balanced blood glucose levels in the body to keep it functioning well. It also secretes enzymes such as lipase, amylase, chymotrypsin, and trypsin to help break down food in the digestive tract for proper digestion and absorption [38-39]. This carcinoma tends to spread fast to other parts of the body, for instance, the lymph nodes, lungs, liver, and bones. The most common place for lesions for adenocarcinoma is in the small ducts and ductules and is then called the pancreatic intraepithelial neoplasia (PanIN) [38].

PC has one of the highest mortality rates. There have been over 44,000 American PC diagnoses in the year 2010 [38-39]. Gender does not have an effect on who would more likely get cancer, but the average age of the development of PAC is above 45 years of age [38].

The pancreas can have cancer cells arising from either the exocrine or endocrine portion, although 95% of them arise from the exocrine portion, which means that the digestive juices that help in absorption and digestion are affected most [38-39]. Tobacco smoking is the most common risk factor for PAC, accounting for about 20% to 25% of PCs [10,38]. Smoking is one of the most common causes of PC, for not only does it increase the risk factor for pancreatic cancer, but it also accelerates the progression for malignancy. Other risk factors include obesity, diets associated with high fat, a history of pancreatitis, and diabetes [39-40].

PAC generally arises due to the consequence of a cancer-associated mutation that is acquired or inherited. There are four confirmed genes most commonly affected, which are the KRAS, cyclin-dependent kinase inhibitor 2A (CDKNA2A)/p16, SMAD family member 4 (SMAD4), and TP53. The cause for these changes is not yet known, but these gene mutations are ways cancer arises and begins to grow [38-40].

PAC usually stays silent until it spreads or extends to impinge on another structure. Symptoms that a patient with PC may present with most likely start with pain in the upper abdomen that may radiate to the back, weight loss, early satiety, depression, and fatigue [38-39]. Some complications of PAC include the development of obstructive jaundice and a Courvoisier gallbladder [39]. Unfortunately, when patients present with these symptoms, the disease progression is quite far, and most are not resectable, resulting in a poor prognosis.

The treatment for PC is primarily surgery and secondly, is chemotherapy. Removal of the cancer cells before metastasis offers the best prognosis. If cancer has spread past the pancreas, then chemotherapy and radiation should be used. Chemotherapy works throughout the body by preventing dividing cells from spreading further by helping to kill them, and radiation helps by the destroying cancer cells using a high energy wave [38,40].

Renal cancers associated with CS

Renal Cell Carcinoma

RCC is the most common type of kidney cancer in adults and has a strong association with smoking. RCC is formed when cancer cells located in the tiny proximal tubules of the kidneys begin to grow uncontrollably. These tubules help with the filtering of blood and getting rid of toxic waste products that will ultimately be excreted as urine [41]. The tubules are lined with a simple cuboidal epithelium and have a border covered with microvilli, and when this area is compromised by RCC, the functions of absorption and secretion are compromised [28,41].

RCC is the most common type of kidney cancer in adults and accounts for 90% to 95% of abnormal tissue growths in the kidney. There have been about 65,000 RCC cases annually, with 13,700 resulting in death [19]. The cases generally peak around the ages of 50-70 years old, and males tend to be affected more than females, although it is not limited to those parameters [19,41]. A risk factor associated with RCC is HTN, which males tend to be at a higher risk to develop. Men are also noted to smoke more than females, and they are more likely to be exposed to cancer-causing chemicals at work, making them at a higher risk of developing RCC [19,42].

The exact etiology as to why RCC form is unknown, although there are many known risk factors, which include smoking, obesity, HTN, a family history, and having advanced kidney disease [19]. The risk from smoking depends on the amount smoked. The chronic smoker will have a greater risk over the occasional smoker to develop RCC. RCC can be described in five different forms, namely the following clear cell carcinoma (CCC), papillary tumors (PTs), chromophobe tumors (CTs), oncocytomas, and collecting duct. Each of these types has specific histological features and distinguishing locations, which differentiates them from one another [19,43]. CCC is the most abundant type, making up for about 60% of the cases, and is located in the proximal tubule. The CCC histologically appears clear and shows an acinar or sarcomatoid cell (SC) growth pattern and tends to have a very good response to treatment [19]. PTs make up 5% to 15% of cases and show a growth pattern of papillary or SCs histologically and are also located in the proximal tubule [19,42]. CTs present as solid or tubular cells histologically, are a less aggressive form of RCC, and make up about 5% to 10% of cases. They are less aggressive because of their large size, compared to the other types, and due to the size, they are not able to spread past the kidneys. CTs are located in the distal tubules of the kidney and the cortical collecting duct. Oncocytomas are present in the cortical collecting ducts and show a nest tumor-type growth, make up 5% to 10% of cases each, and are also a less aggressive form since they tend not to spread past the kidney. Lastly, the rarest and toughest form to treat, making up less than 1% of cases, is collecting duct RCC. It presents with a papillary or SC growth pattern, and it originates in the medullary collecting duct [19].

CS doubles the risk of RCC due to the fact that it slows the blood flow to the kidneys. There is generalized vasoconstriction that ultimately results in the kidneys’ function to be decreased, making them weaker and more susceptible to disease [11]. Smoking also advances the progression of the disease; it makes it faster due to the decreased supply of blood flow [44]. The study has also shown that smoking causes DNA methylation, which also leads to cancer formation [19,43-44].

Patients can present with a wide range of symptoms, the most important being hematuria, abdominal pain, and a mass in the abdominal region. Some other symptoms patients typically present are weight loss, fever, and varicocele [19,44]. The treatment for RCC is mainly surgery but can also include radiation therapy. For patients with localized RCC, a partial or total nephrectomy, depending on the stage or spread of cancer, is used [19]. For patients unable to undergo surgery, radiation is the next considered procedure to best target the small lesions and remove cancer. Patients who have metastasized RCC have a poor prognosis, and treatment usually revolves around targeted therapy and immunotherapy [19,42]. Lastly, chemotherapy is also used if cancer has metastasized to other parts of the body outside the kidney [19,42-43].

Bladder cancer associated with CS

Urothelial Cell Carcinoma

Urothelial cell carcinoma (UCC) is also called transitional cell carcinoma and is the most common urinary tract cancer that is observed in 90% of cases, most commonly arising from the urothelium of the bladder. The “urothelium" is made up of a transitional epithelium cell lining, otherwise known as the mucosal surfaces of the renal collecting tubules, calyces and pelvis, and the ureter, bladder, and urethra [45]. UCC occurs most commonly in the lower urinary tract, the bladder, and the urethra and can also affect the constituents of the upper urinary tract, the renal pelvis of the kidney and the ureter [46].

An estimated 76,960 new diagnoses of BC have been known to arise in the U.S. in 2016, 16,390 of which would result in the death of the patient. Patients who acquire cancer in the upper portion of the urinary tract typically have a 30% to 50% chance of developing cancer in the bladder, while those with BC have only 2% to 3% chance of developing cancer in the upper urinary tract [45]. Men acquire cancer of the urothelial tract at a higher rate than women, with a ratio of 3:1, and a peak incidence for both at age 70 [45,47]. Whiles acquire UCC at a two times higher rate than African Americans [45-46].

CS is the major cause of UCC [12]. A strong correlation has been found between cancers of the urothelial tract at all levels, particularly in relation to the duration and amount of cigarette smoke inhaled. Among new BC diagnoses, 90% of cases occur in current or former smokers. Toxicologists have estimated that over 70 confirmed carcinogenic toxins are present within tobacco smoke [46].

BCs are identified based on how far they invade into the wall of the bladder and are categorized as invasive or non-invasive. Invasive cancers grow into the deeper layers of the bladder wall and are more likely to spread and thus are difficult to treat. Non-invasive cancers remain in the inner layer of the cells, the transitional epithelium, but do not grow into the deeper layers of the bladder wall. Both invasive and non-invasive bladder cancers can be described as superficial, or non-muscle invasive bladder cancer (NMIBC) [45,47].

In about 30% of newly diagnosed urothelial bladder tumors, there are multiple sites of bladder involvement, most commonly as carcinomas in situ. A carcinoma in situ that involves the bladder diffusely, without also having a superficial tumor associated with it, is considered an aggressive disease. Most patients manifesting this combination will develop muscle-invasive bladder cancers. However, when carcinoma in situ is associated with superficial tumors, rates of recurrence and disease progression are higher (50% to 80%) than when no such association is present (10%) [12]. When urothelial tract cancers are staged, superficial BC includes PTs that involve only the mucosa (Ta [low-grade]) or submucosa (T1) and flat carcinoma in situ (Tis [CIS]). The NMIBC accounts for about 80% of all BCs; Ta BC accounts for most of NMIBC (60%) where T1 and Tis account for 30% and 10%, respectively [47].

Aside from invasive and non-invasive, BCs are further subdivided into papillary and flat types. Papillary carcinomas (PTCs) are finger-like projections that grow from the inner surface of the bladder towards its hollow center. The PTs often grow towards the center of the bladder, but do not grow into the deeper bladder layers; they are therefore called non-invasive papillary cancers [48]. When cancer is slow growing, it means that it is very low grade, considered to be a non-invasive papillary carcinoma, and can be called a papillary urothelial neoplasm of low-malignant potential (PUNLMP). This neoplasm tends to have a very good outcome. Flat carcinomas do not grow towards the hollow part of the bladder. If a flat tumor (FT) stays in the inner layer of the bladder cells, it is called a non-invasive flat-carcinoma, or a flat carcinoma in situ (CIS). If a PT or FT grows into the deeper layers of the bladder, then it is called invasive UCC [49].

Conventional urothelial carcinoma (CUC) is the most common neoplasm that affects the urothelial tract. It may be subdivided into low- and high-grade non-invasive lesions, as well as high-grade invasive carcinomas. Invasive high-grade UCC can progress from either flat urothelial carcinoma in situ (flat CIS), or a non-invasive high-grade papillary urothelial carcinoma by a series of molecular alterations, including smoking, chemical and environmental toxins, etc. By contrast, other papillary urothelial neoplasms rarely progress to high-grade lesions, although they still may recur [47-50]. This subtype of neoplasms includes the following: papillary urothelial neoplasm of low malignancy, non-invasive low-grade papillary UCCs, and papillomas. They are thought to arise from different genetic pathways. This is seen when comparing patients with low-grade papillary neoplasms who have a 15% risk of progression to invasion or flat CIS and patients with high-grade papillary neoplasms who contrastingly have a >50% risk of progression [48].

The molecular distinction between low- and high-grade non-invasive urothelial neoplasms, as well as flat CIS, shows a loss of function of a suppressor gene mutation, retinoblastoma 1 (RB1). In high-grade lesions, it is TP53 in which its suppressor function is lost, as well as overexpression of the oncogenes EGFR/Erb-B2 receptor tyrosine kinase 1 (ERBB1) and Erb-B2 receptor tyrosine kinase 2 (ERBB2), and other alterations they exhibit. The changes in the underlying microvasculature are evident at all stages of urothelial carcinogenesis. The following genetic factors mediate changes in microvasculature: vascular endothelial growth factor (VEGF), VEGF receptors, angiopoietins, and thrombospondins. Similar molecular findings found in non-invasive high-grade UCC are also found in invasive UCC, which include alterations in the genes TP53 and phosphatase and tensin homolog (PTEN). Many of the molecular alterations of a variety of tumor suppressor genes/oncogenes and cell adhesion molecules have been examined in both non-invasive and invasive UCC. The molecular alterations to these genes have been described to correlate with an altered prognosis, appearing to hold potential prognostic and therapeutic promise. These include E-cadherin, p53, the RB protein, and the EGFR [12,48].

It is estimated that one-third of BC cases could be prevented through simple modification of lifestyle choices, in particular, the cessation of smoking tobacco products (cigarettes, cigars, pipes, etc.) [46]. The management of UCC depends on these if it is superficial, invasive, and metastatic. The treatment includes resection, radical cystectomy, and chemotherapy. However, even if resected, the risk of recurrence is high [12,46,48]. The prognosis of urethral cancer is poor (five-year survival of ∼ 45%) [49-50].

## Conclusions

The positive correlation between CS and a handful of carcinomas is predominantly due to tobacco smoke’s carcinogenic effect on normal physiology. These carcinogens have been known to lead to DNA damage by impairing the DNA repair mechanism and weakening the immune system, ultimately leading to inflammation and infections. These two pathological consequences of tobacco smoke predispose multiple organs to the development of carcinomas. Although OC and LCs are primarily associated with CS, this study discusses the role in the pathogenesis of additional carcinomas caused by the carcinogens present in the smoke as well. Treatment of the acquired carcinomas is possible by various means depending on the specific carcinoma, although smoking cessation is the most effective means found to prevent the prevalence and incidence of such carcinomas.
